# The Challenge of Tungsten Skarn Processing by Froth Flotation: A Review

**DOI:** 10.3389/fchem.2020.00230

**Published:** 2020-04-16

**Authors:** Yann Foucaud, Lev Filippov, Inna Filippova, Michael Badawi

**Affiliations:** ^1^Université de Lorraine, CNRS, GeoRessources, Nancy, France; ^2^National University of Science and Technology MISIS, Moscow, Russia; ^3^Université de Lorraine, CNRS, Laboratoire de Physique et Chimie Théoriques, Nancy, France

**Keywords:** scheelite, collectors, depressants, fatty acids, calcium minerals

## Abstract

Recently, tungsten has drawn worldwide attention considering its high supply risk and economic importance in the modern society. Skarns represent one of the most important types of tungsten deposits in terms of reserves. They contain fine-grained scheelite (CaWO_4_) associated with complex gangue minerals, i.e., minerals that display similar properties, particularly surface properties, compared to scheelite. Consistently, the froth flotation of scheelite still remains, in the twenty first century, a strong scientific, industrial, and technical challenge. Various reagents suitable for scheelite flotation (collectors and depressants, mostly) are reviewed in the present work, with a strong focus on the separation of scheelite from calcium salts, namely, fluorite, apatite, and calcite, which generally represent significant amounts in tungsten skarns. Albeit some reagents allow increasing significantly the selectivity regarding a mineral, most reagents fail in providing a good global selectivity in favor of scheelite. Overall, the greenest, most efficient, and cheapest method for scheelite flotation is to use fatty acids as collectors with sodium silicate as depressant, although this solution suffers from a crucial lack of selectivity regarding the above-mentioned calcium salts. Therefore, the use of reagent combinations, commonly displaying synergistic effects, is highly recommended to achieve a selective flotation of scheelite from the calcium salts as well as from calcium silicates.

## Introduction: General Context

### Tungsten: an Overview

Tungsten is a transition metal with the symbol W and atomic number 74, part of the same family as molybdenum and chromium. Its name comes from the former Swedish in which scheelite, the calcium tungstate, was named *tungsten*, i.e., “heave stone.” Tungsten displays very interesting properties for industrial applications. First, it is one of the densest metals on Earth with a specific gravity of 19.3, very similar to that of gold and significantly higher than that of other transition metals. Besides, it exhibits a melting point of 3,422°C, which constitutes the highest melting point among all the metals (only exceeded by graphite). Along with its thermal dilatation coefficient, the lowest among all metals, this makes tungsten an excellent refractory material. Furthermore, this latter displays a substantial hardness (7.5 on the Mohs's scale), especially when it is combined with carbide anions in tungsten carbide (WC), which exhibits a hardness of 9. Tungsten presents a low abundance in the Earth crust, around 1.3 ppm, which is significantly lower than the other lithophile elements but about the same as that of tin (2 ppm) and molybdenum (1.2 ppm). Rarely found under its metal form, tungsten is mostly encountered bound to oxygen atoms (tungstate anions), although it can form sulfides.

### Tungsten Properties, Applications, and Prices

The aforementioned interesting properties of tungsten induce a wide range of industrial applications for this metal, including the production of tungsten carbide (55%), tungsten alloys and supra-alloys (25%), metal tungsten (13%), and chemical compounds (7%) (Audion and Labbé, 2012; U. S. Geological Survey, 2019). In particular, cemented tungsten carbide is traditionally used for machining of metallic parts and products where it constitutes the friction parts of drills and mills. Besides, tungsten alloys and supra-alloys are commonly used for refractory metallic pieces in aircraft engines, munitions, or metallurgical furnaces. Since cemented tungsten carbide cannot be recovered, the recycling rate of tungsten is around 20–25%, which represents a considerably low value (Audion and Labbé, 2012) compared to other metals. Moreover, the noticeable properties of tungsten induce a difficult substitution for this element (Audion and Labbé, 2012; U. S. Geological Survey, 2019): it can be substituted with molybdenum, titanium, or niobium in carbides and alloys, while metal tungsten can be replaced with depleted uranium. However, most of these substitutions decrease, rather than replace, the amount of tungsten used, and generally induce health problems (for uranium), increased cost, or significant loss of product performances (Audion and Labbé, 2012; U. S. Geological Survey, 2019).

**Figure 1:**
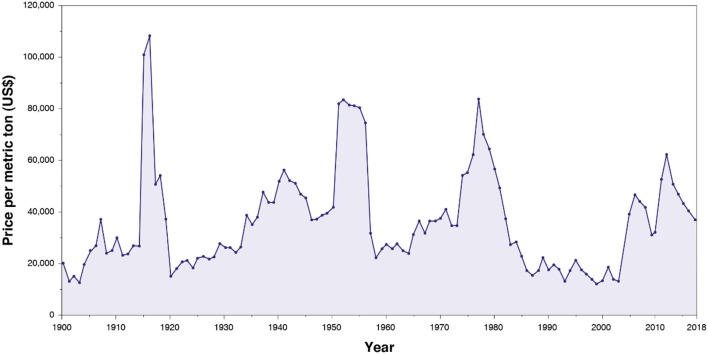
Evolution of the tungsten inflation-adjusted price per metric ton from 1900 to actual, from Metalary website.

Before the twentieth century, tungsten was marginally used for industrial applications. Its consumption significantly rose with the development of heavily armored warships, penetrative ammunitions, and automotive industry at the beginning of the twentieth century. Tungsten inflation-adjusted price has remained constant in average over the twentieth century (Figure 1). Nonetheless, it exhibited considerable peaks (Figure 1), in particular in 1915–1918 (World War I), 1951–1956 (Korean War), 1973–1978, and 2005, with a moderate rise during World War II. In the 1980s, an extra tungsten production ensured by China led to a tremendous decrease in the tungsten price (Audion and Labbé, 2012). Subsequently, most of the European tungsten mines, including the Salau French mine, closed, and very few tungsten mines in the world remained in operation. Since these closures, China has held a monopoly on the global tungsten production and has maintained the tungsten price significantly low. Nevertheless, in 2005, some exportations quotas were applied by China on its tungsten production, inducing a global increase in the tungsten price and, therefore, a gain of interest for tungsten exploration in the rest of the world, including in the EU.

### Tungsten Deposits, Resources, and Supply

Several tungsten minerals have been reported in the literature, but only scheelite (CaWO_4_) and wolframite [(Fe,Mn)WO_4_] are of economic importance and nowadays exploited for primary tungsten extraction (Pitfield et al., 2011; Audion and Labbé, 2012; Schmidt, 2012a,b; Yang, 2018). Wolframite is a continuous solid series between ferberite (FeWO_4_) and hubnerite (MnWO_4_); the Fe/Mn ratio in the mineralized rock defines the dominance of one on another. Four major types of tungsten ore deposits have been reported in the literature: skarns, veins/stockworks, porphyries, and stratabound deposits (Werner et al., 1998; Schubert et al., 2006; Pitfield et al., 2011; Jébrak et al., 2016). As an overview, the typical WO_3_ grades, deposit sizes, tungsten-bearing mineral(s), and mines corresponding to the four major types of tungsten ore are summarized in Table 1. Overall, skarn ores exhibit low tonnages and moderately high grades compared to the three other tungsten deposits, mainly the porphyry deposits.

**Table 1:** The four major types of tungsten ore deposits with typical deposit sizes, WO_3_ grades, tungsten minerals and mines, adapted from Werner et al. (1998), Schubert et al. (2006), Pitfield et al. (2011), and Yang (2018).

**Deposit type**	**Deposit sizes**	**Typical grade, %WO_**3**_**	**Tungsten mineral(s)**	**% of total**	**Mines**
Skarn	10^4^-5 × 10^7^ t	0.3–1.4	Scheelite	41	Cantung (Canada); Los Santos (Spain); Vostok-2 (Russia)
Vein/stockwork	10^5^-10^8^ t	Variable	Wolframite	35	Pasto Bueno (Peru); Panasqueira (Portugal); San Fix (Spain); Chollja (Bolivia);
Porphyry	10^7^-10^8^ t	0.1–0.4	Wolframite Scheelite	16	Xingluokeng (China); Yangchuling (China); Northern Dancer (Canada); Climax (USA)
Stratabound	10^6^-10^7^ t	0.2–1.0	Scheelite	3	Mittersill (Austria); Damingshan (China); Mount Mulgine (Australia)

**Figure 2:**
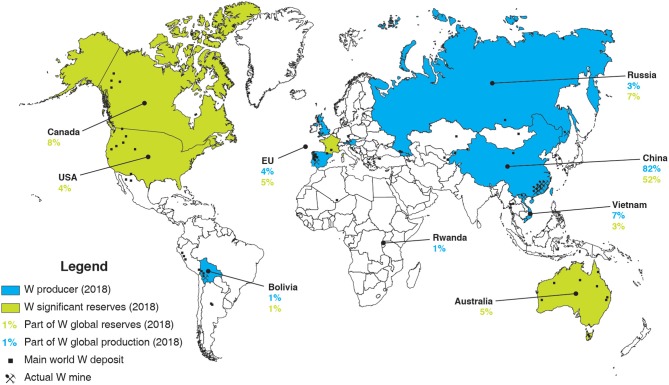
World map of main W world deposit, W mines, W producers and countries with significant W reserves, and their respective part in the global W reserves/production in 2018, built with data from the U. S. Geological Survey (2019).

At the moment, the world tungsten production is mainly ensured by China, which produced, in 2018, more than 80% of the 82,000 t of tungsten produced worldwide (U. S. Geological Survey, 2019). More than 10 major tungsten mines, with an annual output of over 1,300 t of WO_3_, are reported in China, most of them being located in southern China (Werner et al., 1998; Pitfield et al., 2011; Audion and Labbé, 2012; Yang, 2018). In particular, the Xianglushan and Shizhuyuan deposits represent the two largest tungsten mines in China, with over 5,700 and 5,500 t of WO_3_ produced each year, respectively (Yang, 2018). Some other countries such as Vietnam, Russia, and a few European countries produce low amounts of tungsten (Figure 2). Vietnam operates one of the largest tungsten mine worldwide, the Nui Phao mine, whose reserves have been estimated to 66 million tons of ore with an average grade of 0.2% WO_3_ (Masan Resources, 2012). Besides, Russia has been mining the Vostok-2 sulfide–scheelite skarn ore since 1969, with around 1 million tons of remaining ore with a high average grade of around 1.7% WO_3_ (Soloviev and Krivoshchekov, 2011). Despite the decrease in tungsten price in the 1980s, Austria and Portugal succeeded in maintaining the Mittersill and Panasqueira mines in operation. They produced, in 2018, 980 and 770 t of tungsten, respectively, which, however, represent a very minor part of the world production (Figure 2). Recently, considering the significant increase in tungsten price in the middle of the 2000s, new tungsten mining projects have been launched in the world, including in the EU (Suárez Sánchez et al., 2015). In particular, the Los Santos mine (Spain) started operations in 2008 and produced, in 2018, 750 t of tungsten, with estimated reserves of 3.58 million tons with an average grade of 0.23% WO_3_ (Wheeler, 2015). Furthermore, operations started in the Hemerdon mine (UK) in 2015, which reserves have been estimated at 35.7 million tons of ore at 0.18% WO_3_ (Yang, 2018). Nonetheless, despite a production of 900 t of tungsten in 2018, the Hemerdon mine ceased trading operations in October 2018, mainly due to a decrease in tungsten price along with poor processing performances.

**Figure 3:**
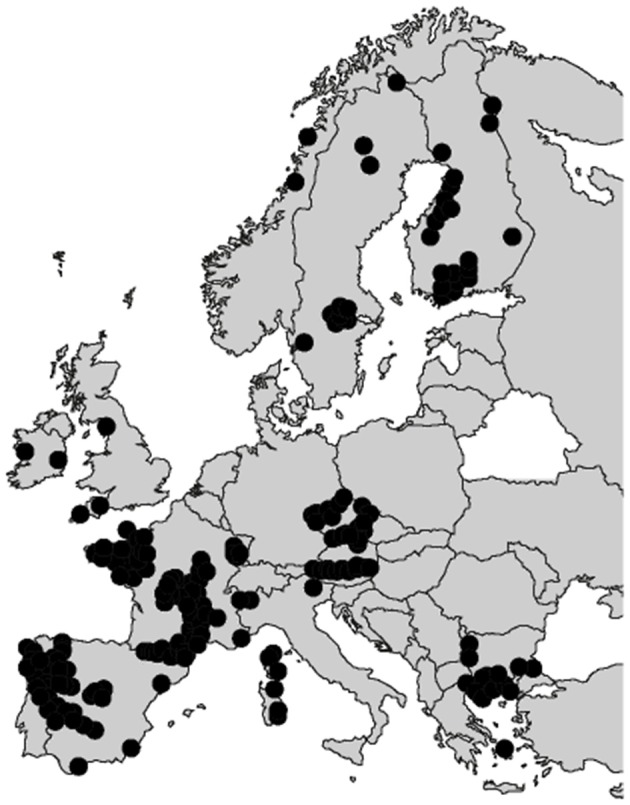
Reported tungsten occurrences in the EU according to the main databases. Adapted from Lauri et al. (2018).

Overall, the EU tungsten production amounts to <3,000 t, while its consumption is estimated at about 10,000 t per year, with a slight continuous increase predicted for the next decade (Yang, 2018). Increasing the tungsten primary production as well as the tungsten recycling rate is, therefore, crucial to afford the EU independency in terms of tungsten consumption. Nowadays, the global tungsten reserves and resources are estimated at 3.3 million tons (U. S. Geological Survey, 2019) and 4.0 million tons of tungsten (Pitfield et al., 2011; Yang, 2018), respectively, with around 52% in China (Figure 2). Nevertheless, many tungsten occurrences have been reported in the EU, including the UK, Spain, Austria, Portugal, and France (Figure 3), some of which exhibiting significant resources and being suitable for exploitation.

Based on the previous considerations, tungsten has been classified as a critical raw material (CRM) in the EU since 2011. Hence, some research programs have been launched over the past few years to stimulate the tungsten extraction, such as the FAME (Flexible and Mobile Economic Processing Technologies) H2020 project. In particular, the stress has been put on tungsten skarns, which represent, within the four major tungsten deposit types, more than 40% of the global tungsten reserves (Werner et al., 1998; Schubert et al., 2006; Pitfield et al., 2011). However, despite significantly high WO_3_ grades, most skarn ores are still considered complex for mineral processing: the development of efficient, environment-friendly, and mobile processing routes for tungsten skarns beneficiation is therefore of paramount interest.

### Tungsten Skarns

#### What Is a Skarn?

**Figure 4:**
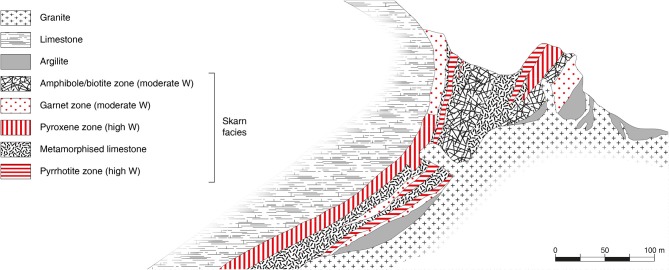
Cross-section of a typical tungsten skarn (Cantung skarn, Canada) showing the granite, the limestone, and the different skarn facies at their interface, including the tungsten-bearing lithologies. Modified from Dawson (1996) with permission from the Geological Survey of Canada.

Skarn deposits are one of the most abundant ore types in the Earth's crust and have been intensively studied over the past decades (Meinert et al., 2005). Around 150 publications per year contain the term “skarn,” which indicates a high constant interest of researchers and industrials for such deposits. Initially, skarns were defined by their mineralogical composition, usually dominated by calc-silicates such as pyroxene or garnets, the formation of which is enabled by the significant amounts of calcium in protolithic rocks (Einaudi and Burt, 1982; Kwak, 1987; Dawson, 1996; Misra, 2000; Meinert et al., 2005). However, skarns can also be considered in a more broad way, as rocks produced by the replacement of calcite or dolomite marble regardless of the presence of calcic or magnesian silicates (Kwak, 1987). Skarn deposits occur throughout a wide range of geological backgrounds, from Precambrian to Cenozoic, although most economic deposits are young and associated with magmatic–hydrothermal activity related to plutonism in orogenic belts (Einaudi and Burt, 1982). Traditionally, skarns are formed during contact metamorphism that goes along with a variety of metasomatic processes involving fluids of magmatic, metamorphic, meteoric, and/or marine origin (Meinert et al., 2005). Hence, most skarns are encountered adjacent to plutons albeit they can occur along faults, major shear zones, and various other structural backgrounds (Figure 4). Skarns can be classified by considering several major criteria, e.g., their calcite/dolomite initial content (calcic vs. magnesian skarns), their Fe^3+^/Fe^2+^ ratio (oxidized vs. reduced skarns), or their distance to the pluton (proximal vs. distal skarns), to name but a few (Einaudi and Burt, 1982; Kwak, 1987; Meinert et al., 2005). Overall, though some skarns occur in not-calcic rocks, most of economic tungsten skarns are hosted in rocks with significant calcium contents (and/or magnesium) since this element is essential for scheelite deposition in the ore. This induces a common dominance of calcium minerals associated with scheelite in such ores.

#### The Economic Importance of Skarns

**Figure 5:**
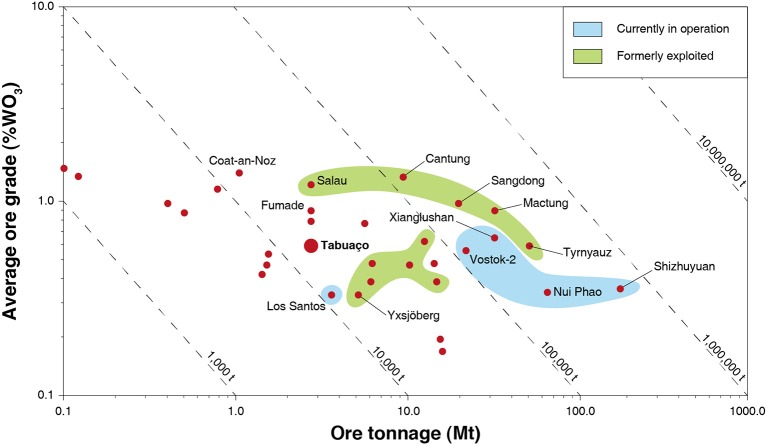
Grade vs. tonnage of significant tungsten skarn deposits in the world, currently or formerly in operations. Modified and updated from Dawson (1996) with permission from the Geological Survey of Canada.

Mining of skarn deposits dates back to at least 4,000 years, and clear evidences of the mining of skarns can be encountered in the ancient Chinese, Greek, and Roman empires (Meinert et al., 2005). Historically, skarns have been mined for a large variety of metals, including iron, tin, tungsten, copper, zinc (along with lead), molybdenum, and gold (Einaudi and Burt, 1982; Kwak, 1987; Meinert, 1992; Dawson, 1996; Meinert et al., 2005). Considering actual economic deposits, skarns display either too low grades (for iron) or tonnages, e.g., for lead, zinc, or copper (Kwak, 1987) and, therefore, are no more exploited. Nonetheless, tungsten skarns commonly display average WO_3_ grades ranging from 0.3 to 1.5%, which are significantly higher than for the other major types of tungsten deposits (see Tungsten Deposits, Resources and Supply section) (Table 1 and Figure 5). Hence, despite lower tonnages compared to other major types of tungsten deposits (Table 1), many tungsten skarns are of economic importance. They have been continuously exploited for decades (Figure 5), supplying most of the world's tungsten demand (>70%) during some periods such as in the 1980s (Kwak, 1987). Nowadays, China ensures 82% of the world tungsten production (U. S. Geological Survey, 2019) with a considerable part of the Chinese tungsten coming from skarn deposits (Audion and Labbé, 2012). Indeed, the Xianglushan and Shizhuyuan world class tungsten deposits, which account for more than 11,000 tons (14%) in the annual global tungsten production, are considered as tungsten skarns (Lu et al., 2003; Cheng, 2016; Dai et al., 2018). Additionally, the Nui Phao and Vostok-2 mines, which together produce 10% of the world tungsten, are also exploiting tungsten skarns (Soloviev and Krivoshchekov, 2011; Masan Resources, 2012) Hence, tungsten skarns represent a significant part in the current tungsten production, and some authors estimate that they account for more than 40% of the global tungsten reserves (Werner et al., 1998; Schubert et al., 2006; Pitfield et al., 2011). Besides, many tungsten skarns have been reported in the world, including in the EU; some are currently exploited, while the other largest ones were exploited in the second half of the twentieth century (Figure 5).

#### Processing Problematics and Options

Considering the high economic potential of tungsten skarn ores, the classification of tungsten as a CRM resulted in a global resurgence of interest for these ores (Suárez Sánchez et al., 2015; Kupka and Rudolph, 2018a; Yang, 2018). However, most authors working on tungsten skarns, either in the geology or in the mineral processing fields, have noticed complex gangues for the tungsten extraction, in particular for the ore dressing stage. Indeed, skarns generally occur in limestone protoliths, which induce high amounts of calcium and, therefore, the formation of calcium-bearing minerals. Owing to these metallogenetic processes, scheelite is found in fine-grained mineralization disseminated thorough the ore body and commonly associated with calcium silicates such as garnet, pyroxene, epidote, vesuvianite, wollastonite, etc., as well as with calcium salts such as fluorite (CaF_2_), apatite [Ca_5_(PO_4_)_3_(OH,Cl,F)], and calcite (CaCO_3_) (Einaudi and Burt, 1982; Kwak, 1987; Meinert, 1992; Dawson, 1996; Lu et al., 2003; Meinert et al., 2005; Cheng, 2016; Jébrak et al., 2016; Dai et al., 2018).

The aforementioned gangue calcium-bearing minerals comprise elements such as P, Si, C, and F that are considered (along with sulfur) as penalizing elements either for the scheelite concentrates processed by hydrometallurgy or for the final metal–tungsten product (Pastor, 2000; Pitfield et al., 2011; Yang et al., 2016). Moreover, tungsten is traditionally extracted from scheelite concentrates by the hydrometallurgy process that requires >60% WO_3_ in the concentrates (Pastor, 2000; Pitfield et al., 2011), while skarn ores usually assay between 0.5 and 1.5% WO_3_ (Figure 5). Hence, removal of fluorite, apatite, calcite, and calcium-bearing silicates prior to any hydrometallurgical treatment is mandatory. The development of an efficient mineral processing flowsheet for the rejection of the abovementioned minerals from a given skarn ore allows to make this skarn of economic potential, i.e., exploitable.

The beneficiation of scheelite ores generally consists of crushing and grinding, followed by successive purification stages to produce a concentrate assaying 65-75% WO_3_ to meet the product specifications imposed by the international trading (Lassner and Schubert, 1999; Pastor, 2000). As wolframite, scheelite is brittle and, therefore, tends to form fine particles during the milling stage. Traditionally, the comminution is carefully designed to avoid overgrinding with regular and appropriate classifying stages all over the process. In terms of processing, scheelite is diamagnetic as most of the gangue minerals, including fluorite, apatite, calcite, and some silicates such as quartz, feldspars. Therefore, magnetic separations are most of the time unsuitable for scheelite ores. Second, scheelite exhibits a significant specific gravity (6.1), which enables the use of gravity separations for scheelite processing. However, skarn ores generally display fine liberation sizes at which the classical gravity separation apparatuses are poorly efficient, inducing high WO_3_ losses. In addition, the losses during the physical separation stages are significantly increased by the trend of scheelite to form fine particles (<10 μm), which are known to be lost in such separations. Consequently, scheelite has traditionally been beneficiated by froth flotation since the 1930s, when this process became a powerful route for fine particle processing. When the liberation size allows it, skarn ores can be processed by a combination of gravity separation and froth flotation. Scheelite flotation was thoroughly investigated during World War II in the USA and USSR as the tungsten demand was significantly high for military applications. However, scheelite flotation has been widely spread in the 1970s, which resulted in a sudden increase in the scientific interest for scheelite flotation and, hence, in the publication of many studies dealing with this topic (Kupka and Rudolph, 2018a). Owing to the depletion of wolframite reserves in China along with the European Metal policy, scheelite flotation has gathered considerable interest in the world over the past few years (Pitfield et al., 2011; Suárez Sánchez et al., 2015; Kupka and Rudolph, 2018a; Yang, 2018). Nonetheless, skarn deposits commonly exhibits complex gangues (Kwak, 1987; Dawson, 1996; Misra, 2000; Meinert et al., 2005; Jébrak et al., 2016), i.e., gangue minerals that display similar surface properties to that of scheelite. Hence, although the two main processing options, namely, gravity separation and froth flotation, can be suitable for tungsten skarns, they have to be thoroughly investigated and finely adapted to the rock characteristics, particularly the froth flotation process.

## The Froth Flotation, A Mandatory Stage in the Scheelite Processing?

### Surface Properties of Scheelite

**Figure 6:**
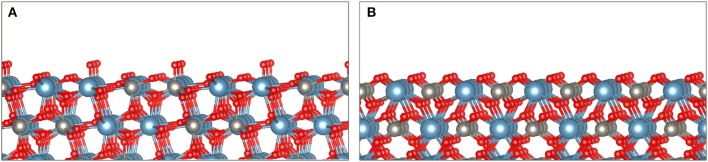
The two main exposed surfaces during scheelite cleavage, namely, the (112) surface **(A)** and the (001) surface **(B)**, based on the literature cited in the text. These surfaces exhibit two dangling bonds per calcium atom, while each calcium atom is eight coordinated in the scheelite lattice.

Scheelite is an ionic mineral composed of Ca^2+^ cations and ${\text{WO}}_{4}^{2-}$ anions, occurring in the I4_1_/a space group (tetragonal system). The most exposed surfaces have been extensively studied by means of atomistic calculations as well as of various experimental methods. Scheelite cleaves mostly forming the (112) and (001) surfaces since they present the lowest surface dangling bond densities (Mogilevsky et al., 2004; Hu et al., 2012; Gao et al., 2013, 2016b; Li and Gao, 2017). The average W–O bond length is 1.777 Å compared to 2.458 Å for the Ca–O bond length. Consistently, this induces significant differences in bond energies between W–O (610 kJ mol^−1^) and Ca–O (130 kJ mol^−1^) (Neiman, 1996). Hence, during the cleavage process, most ${\text{WO}}_{4}^{2-}$ anions remain intact, while the Ca–O bonds break, resulting in a surface constituted of large polyatomic ${\text{WO}}_{4}^{2-}$ anions bonded with Ca^2+^ cations (Figure 6). The (001) and (112) cleavage surfaces exhibit two dangling bonds per calcium atom (Figure 6). As each calcium atom is eight coordinated in the scheelite lattice, the (001) and (112) surfaces comprise six-coordinated calcium atoms, which results in a significant reactivity of the surface calcium atoms.

Considering the significant electronegativity difference existing between ${\text{WO}}_{4}^{2-}$ and Ca^2+^, scheelite is a sparingly soluble polar salt-type mineral. Hence, it displays a significant free energy value at its surface, which induces a favored adsorption of water molecules or hydroxyl anions on its surface, making it significantly hydrophilic (Hu et al., 2012; Wills et al., 2016; Gao et al., 2016b). Some authors have investigated the solubility of scheelite in water at room temperature (25°C): the solubility product remains roughly constant at pH > 6 with a value of 8.9 × 10^−9^ (Marinakis and Kelsall, 1987a) or 4.9 × 10^−10^ (Atademir et al., 1979), which substantiates the semi-soluble behavior. The solubility increases significantly below pH 6 (Arnold and Warren, 1974; Atademir et al., 1979; Marinakis and Kelsall, 1987a), which can be mainly attributed to the displacement of the dissolution equilibrium by the formation of isopolytungstate species (polymerized tungstate) in solution (Marinakis and Kelsall, 1987a). Interestingly, the molar concentration of ${\text{WO}}_{4}^{2-}$ above pH 6 is significantly higher than that of Ca^2+^, resulting in an excess of ${\text{WO}}_{4}^{2-}$ anions near the surface, i.e., in the inner plane. This explains the negative zeta potential of scheelite over the whole pH range (Arnold and Warren, 1974; Atademir et al., 1979; Marinakis and Kelsall, 1987a; Hicyilmaz and Özbayoglu, 1992; Ozcan and Bulutcu, 1993; Gao et al., 2016b), although high Ca^2+^ concentrations can conduct to a zero zeta potential. This is attributed to increased amounts of Ca^2+^ ions in the inner plane that balance the ${\text{WO}}_{4}^{2-}$ provided by the scheelite dissolution (Atademir et al., 1979; Hicyilmaz and Özbayoglu, 1992).

### Collection of Scheelite

Since scheelite is a polar mineral, water molecules are adsorbed as soon as surfaces are generated during the milling stage, making them hydrophilic. Hence, it is required to add flotation collectors to make scheelite hydrophobic and recover it in the froth (Bulatovic, 2007). Two main surface properties can be exploited for the adsorption of flotation collectors onto scheelite: the existence of undercoordinated surface calcium atoms (Mogilevsky et al., 2004; Hu et al., 2012; Gao et al., 2013, 2016b; Li and Gao, 2017), i.e., with two dangling bonds (Figure 6) and the negative zeta potential of scheelite surfaces over the whole pH range (Arnold and Warren, 1974; Atademir et al., 1979; Marinakis and Kelsall, 1987a; Hicyilmaz and Özbayoglu, 1992; Ozcan and Bulutcu, 1993; Gao et al., 2016b). These two properties suggest the use of anionic and cationic collectors, respectively, which can both be employed for scheelite flotation.

#### Anionic Collectors

**Figure 7:**
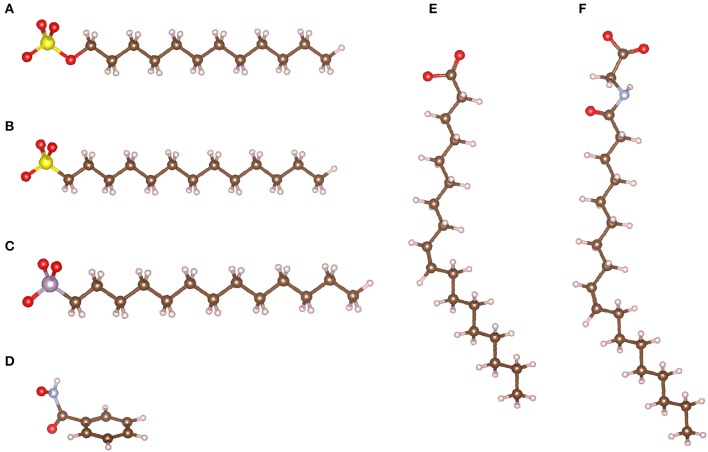
The different anionic collector families used for scheelite flotation: sulfates **(A)**, sulfonates **(B)**, phosphonates **(C)**, hydroxamates **(D)**, carboxylates **(E)**, and sarcosinates **(F)**. The maroon, white, red, yellow, purple, and blue-gray balls represent the carbon, hydrogen, oxygen, sulfur, phosphorous, and nitrogen atoms, respectively.

Anionic collectors are composed of a polar group exhibiting a negative charge available to establish a chemical bond with undercoordinated surface cations of many minerals, including scheelite. Hence, anionic collectors are widely used for a large range of minerals (Leja, 1981; Bulatovic, 2007). Notably, the negatively charged atoms of the polar group are traditionally adapted to the target mineral: oxygen atoms (oxhydril) for oxide minerals and sulfur atoms (sulfhydryl) for sulfide minerals (Fuerstenau and Healy, 1972; Fuerstenau and Palmer, 1976; Leja, 1981; Bulatovic, 2007). Therefore, oxhydril collectors are generally used for scheelite (Figure 7), which is an oxide mineral. Most oxhydril collectors correspond to the basic form of an acid–base couple, since the existence of an extra valence electron, i.e., a negative charge, located on the polar group of the collector is induced by the deprotonation of the acidic form.

##### Sulfates, phosphates, and their derivatives

Some authors have investigated the use of sulfate collectors such as sodium dodecyl sulfate (SDS) for scheelite flotation (Grosman and Sukhoval'skaya, 1955; Grosman, 1962; Atademir et al., 1981; Marinakis and Kelsall, 1987b). Nevertheless, they reported a significant lack of selectivity between scheelite and other calcium minerals such as calcite (Atademir et al., 1981; Marinakis and Kelsall, 1987b). The adsorption is related to an exchange between the tungstate anion, ${\text{WO}}_{4}^{2-}$, and the sulfate collector, on the surface (Atademir et al., 1981), supported by the solubility results previously discussed (Atademir et al., 1979; Marinakis and Kelsall, 1987a). Albeit these collectors have been poorly investigated for scheelite flotation over the past decades, they have been extensively studied for other calcium salts such as fluorite. Interestingly, SDS provided high performances for fluorite flotation (Sørensen, 1973), which are mainly attributed to a chemisorption onto surface calcium atoms (Shergold, 1972; Sørensen, 1973; Mielczarski et al., 1983; González-Martín et al., 1996). Furthermore, the calcium sulfate salts exhibit a significant solubility compared to other anionic collectors salts, indicating that no precipitation occurred at the studied concentrations (Fuerstenau and Palmer, 1976). Hence, such collectors are promising despite the crucial lack of selectivity displayed throughout the flotation tests. Besides, sulfonate collectors, which display a chemical structure very similar to that of sulfate collectors (Figure 7), demonstrated acceptable performances for calcium mineral flotation, including scheelite (Fukazawa, 1977; Gao et al., 2015a) and fluorite (Zheng et al., 2018; Chen et al., 2019). However, as for sulfates, sole sulfonates provided poor selectivity between scheelite and other calcium minerals despite good recoveries (Gao et al., 2015a).

In phosphonate molecules, the sulfur atom from a sulfonate is replaced with a phosphorous atom in the polar group (Figure 7). Nonetheless, while sulfur establishes two double bonds with the surrounding oxygen atoms, phosphorous forms only one double bond (Figure 7C). As for sulfates, phosphonates are used in basic conditions to favor the anionic form, i.e., the phosphonate, rather than the acidic form (Marinakis and Kelsall, 1987b). The adsorption studies conducted with phosphonate suggested that a chemisorption occurs between the polar group and the surface calcium atoms of scheelite and calcite (Marinakis and Kelsall, 1987b). However, considering the very close calculated adsorption energies of phosphonate on scheelite (−42.9 kJ.mol^−1^) and on calcite (−38.8 kJ.mol^−1^), the adsorption selectivity and, therefore, the flotation selectivity are traditionally very low using those collectors (Marinakis and Kelsall, 1987b).

##### Hydroxamates

Hydroxamates, the anionic forms of hydroxamic acids, have been significantly used for two decades, mainly because of their noticeable chelating abilities. Basically, their polar group contain a carbon atom linked to an oxygen and a nitrogen, these latter being linked to an oxygen atom, which can be both deprotonated to form the anionic forms (Figure 7D). The use of hydroxamic acids has been deeply investigated in the literature by many research articles published between 2000 and 2019 (Pradip and Fuerstenau, 1983, 1985; Zhao et al., 2013, 2015; Feng et al., 2017; Han et al., 2017; Tian et al., 2018; Wei et al., 2018, 2019; Yue et al., 2018). First, Zhao et al. (2013) demonstrated that cyclohexylhydroxamate (CHA) exhibits performances significantly better than benzohydroxamate (BHA) for scheelite collection (Zhao et al., 2013). Nonetheless, the flotation recovery was not as high as other collectors such as fatty acids and hydroxamates commonly suffer from a lack of selectivity (Zhao et al., 2013). Hence, most studies have shown that BHA should be combined with lead ions to increase notably the flotation selectivity, providing an acceptable separation of scheelite from gangue minerals, including the calcium salts (Zhao et al., 2015; Feng et al., 2017; Tian et al., 2018; Wei et al., 2018, 2019). The activation mechanism is mainly related to the specific adsorption of Pb^2+^ ions onto scheelite surfaces, since the lead tungstate species are known to be thermodynamically stable. In addition, the chelation ability of BHA is significantly higher for lead ions compared to calcium ions, especially because these latter are included in the crystallographic structure.

##### Sarcosinates

The acyl sarcosinates represent another family of collectors that were developed in the early 1990s by the chemical reaction between a fatty acid and an amino-acid (Schubert et al., 1990). They exhibited very good performances for fluorite flotation from calcite-rich gangues, albeit some specific depressants were used to depress calcite (Schubert et al., 1990). The oleoyl sarcosine (Figure 7F), which is synthesized from oleic acid, also displayed satisfactory performances for scheelite flotation from a siliceous gangue (Ozcan and Bulutcu, 1993; Ozcan et al., 1994). Nevertheless, this molecule provided a poor selectivity between scheelite and calcite, and modifiers were added in a first stage of conditioning to reach an acceptable separation. This method was applied to an ore assaying 0.3% WO_3_, which was first conditioned with 400 g/t of alkyloxine (a hydroxyquinoline) at pH 8, then with 50 g/t of quebracho to depress calcite, and finally with 200 g/t of oleoyl sarcosine as scheelite collector (Ozcan et al., 1994). This flowsheet afforded good separation performances: these authors obtained a concentrate assaying 56.1% WO_3_ with 79.4% WO_3_ recovery. Nonetheless, the ore considered by Ozcan et al. (1994) comprised only calcite as calcium salt, strongly depressed by the use of quebracho. In addition, sarcosinates have been successfully used for fluorite collection (Schubert et al., 1990), indicating a significant affinity of these collectors for fluorite, as well as for scheelite (Ozcan et al., 1994). This is mainly due to the adsorption mechanisms, which are reported to be very similar to that of fatty acids since the polar group of sarcosinates contain a carboxylate group (Xian-Ping et al., 2017).

##### Carboxylates

**Figure 8:**
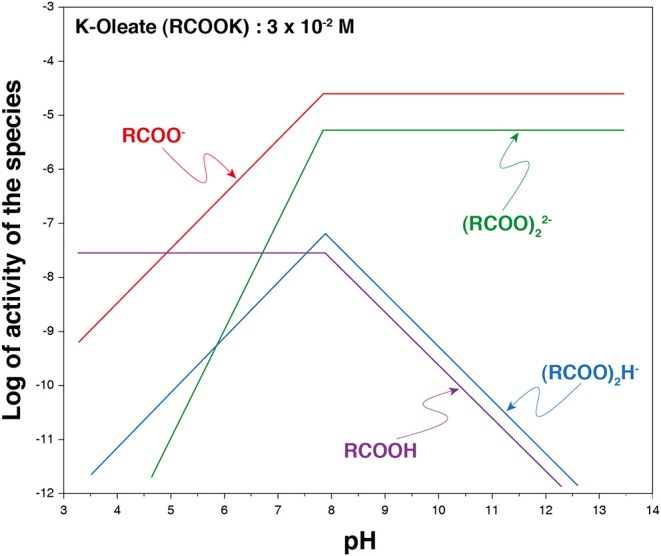
Species distribution diagram for an aqueous (potassium) oleate solution. Adapted from Miller and Misra (1984).

Carboxylates, which correspond to the anionic form of carboxylic acids (Figure 7E), are the most used collectors worldwide for the collection of a large range of oxide (and fluoride) minerals, including silicates and sparingly soluble minerals. These latter are composed of an alkaline-earth, a lanthanide, or a transition metal associated with a mono- or a polyatomic anion. On the one hand, the valuable metal can correspond to the cation, as is the case for rare earth elements [in monazite (La,Ce,Nd)PO_4_ or in bastnäsite (La,Ce,Nd)CO_3_], barium (in barite, BaSO_4_), orstrontium (in celestite, SrSO_4_). On the other hand, the valuable metal can correspond to the cation that forms the polyatomic anion, as niobium, tantalum, tungsten, or boron, which constitute niobate-/tantalite-based minerals [e.g., columbite (Fe,Mn)Nb_2_O_6_], tungstate-based minerals (e.g., scheelite and wolframite), and borate-based minerals (e.g., colemanite, Ca_2_B_6_O_11_, 5 H_2_O), respectively. Besides, the metallic commodity can be the whole anion of the semi-soluble salt such as F^−^ and ${\text{PO}}_{4}^{3-}$, extracted from fluorite and apatite, respectively. All the above mentioned minerals have been successfully collected by carboxylates (Fuerstenau and Palmer, 1976; Leja, 1981; Bulatovic, 2010; Wills et al., 2016) as well as many silicates (Fuerstenau and Palmer, 1976), including andalusite/kyanite (Jin et al., 2019), spodumene (Xu et al., 2016, 2017a; Zhu et al., 2018), feldspars (Xu et al., 2016, 2017a,b), activated quartz (Gaudin and Fuerstenau, 1956; Fuerstenau and Palmer, 1976), muscovite (Alekseev and Morozov, 1975), and beryllium silicates (Walsh and Vidal, 2009). In addition, pure oxides such as cassiterite (Angadi et al., 2015) or hematite (Nakhaei and Irannajad, 2018) have been collected using fatty acids with satisfactory performances. These collectors have been commonly used for scheelite collection considering their low cost, high efficiency, and environment friendliness. The high collection ability of carboxylates for a large range of minerals is mainly related to their significant affinity for metallic cations (alkali-earth, lanthanide, or transition metals). Traditionally, carboxylate collectors are called fatty acids since they are composed of a carboxylic group and a linear aliphatic chain with between 12 and 18 carbon atoms that frequently comprises unsaturations (Leja, 1981). They occur naturally under their acidic form in many vegetal organisms such as trees (mostly pine) or oleaginous plants (olive, colza, peanuts, etc.), which store their energy in the form of lipid molecules. For their use in the froth flotation process, fatty acids are usually treated with a strong base (sodium hydroxide or potassium hydroxide) to form carboxylates anions (metal soaps). The fatty acid deprotonation results in a significant increase in their solubility: authors reported higher solubility products of metal soaps in solution compared to fatty acids, these latter being nearly zero at room temperature (Leja, 1981; Khuwijitjaru et al., 2002), although a significant decrease in the solubility of carboxylate salts occurs when the chain length increases (Fuerstenau and Miller, 1967). Besides, the deprotonation allows adding an extra valence electron, i.e., a negative charge, on the polar group (–COO^−^), which enables the chemisorption of the carboxylate onto the surface metallic cations (Hanumantha Rao and Forssberg, 1991; Foucaud et al., 2018b). In solution, four major species have been reported for the oleic acid/oleate species, depending on the pH and the total concentration (Figure 8). They can be under RCOO^−^, ${\left(\text{RCOO}\right)}_{2}^{2-}$, (RCOO)_2_H^−^, and RCOOH forms, the two first being the most dominant at alkaline pH and for usual concentrations in flotation (Miller and Misra, 1984).

For one century, the *cis*-octadec-9-enoic acid, also named oleic acid, has been the most used carboxylic acid in the flotation worldwide (Leja, 1981; Bulatovic, 2007; Kupka and Rudolph, 2018a), for its high natural abundance and its low melting point (induced by its unsaturation). Hence, it displays low operating costs and considerable ease to work with. Nevertheless, many other fatty acids, with different chain lengths and unsaturation degrees, are used, most of the time in mixtures with oleic acid. Indeed, this latter is difficult to purify from vegetal extracts (trees or oleaginous compounds) and is, therefore, commonly associated with other abundant fatty acids such as the saturated lauric acid (12 carbon atoms), myristic acid (14 carbon atoms), palmitic acid (16 carbon atoms), and stearic acid (18 carbon atoms), or the unsaturated linoleic acid (18 carbon atoms, two unsaturations) and linolenic acid (18 carbon atoms, three unsaturations). Thus, most fatty acid-based collectors are mixtures of the aforementioned fatty acids in different proportions. In particular, tall oil Fatty Acids (TOFA) are fatty acid mixtures derived from the saponification and distillation of pine resin (Logan, 1979). These by-products of the Kraft process in paper industry represent a significant amount of the fatty acids used for froth flotation at industrial scale. In such collector's mixtures, the classical above-described fatty acids are also associated with rosin acids, large-sized hydrophobic terpene-derived compounds among which abietic, pimaric, and palustric acids are dominant (Logan, 1979). Albeit authors suggested that rosin acids do not adsorb onto mineral surfaces (Pearse, 2005), recent studies highlighted their negative impact on the flotation of sparingly soluble minerals: they increase the recoveries of all the minerals therefore inducing a considerable decrease in selectivity (Filippov et al., 2018).

#### Cationic Collectors

Considering the high negative zeta potential of scheelite on the whole pH range, cationic collectors have a high potential for scheelite collection since they are known to adsorb through electrostatic interactions. Among the cationic collectors, amines are the most commonly used for the collection of silicates, oxides, and various other minerals including scheelite. In the literature, some authors investigated the use of amines for scheelite flotation. In particular, Arnold et al. (1978) studied the floatabilities of scheelite and calcite with amines by microflotation as well as their separation by microflotation tests performed on mixtures. They concluded that, despite the good floatabilities of each mineral, the flotation separation of scheelite from calcite remained very difficult using amines (Arnold et al., 1978). In addition, Atademir et al. (1981) demonstrated very good scheelite recovery using dodecylamine hydrochloride which, however, was not suitable for industrial application due to the high silicate contents in traditional ores (Atademir et al., 1981). Later on, Hiçyìlmaz et al. (1993) tested several different amines and showed that amine D acetate (a dodecylamine neutralized with acetate radicals) provided acceptable scheelite recoveries (91%) but poor selectivity between scheelite and calcite (Hiçyìlmaz et al., 1993). Recently, Gao et al. (2015b) investigated the use of dodecylamine (DDA) for the flotation separation of scheelite from calcite with a strong focus on adsorption mechanisms. Interestingly, they showed that, at low pH, electrostatic bonds are established between positively charged amine (${\text{NH}}_{3}^{+}$ head group) and ${\text{WO}}_{4}^{2-}$ surface sites (Gao et al., 2015b). Also, DDA adsorbs better on scheelite than on calcite by creating a more compact monolayer, therefore enhancing the contact angle of scheelite (Gao et al., 2015b).

Besides, the use of quaternary ammonium salts (QAS) as collectors for scheelite has also been investigated (Hu et al., 2011; Yang et al., 2015). Such molecules afforded good scheelite flotation performances along with very good selectivity between scheelite and other calcium minerals such as calcite. For instance, dioctyl dimethyl ammonium bromide was used on a mixture of calcite and scheelite at pH 8 and exhibited high selectivity as well as high scheelite recovery, which were significantly better than those obtained with oleic acid (Hu et al., 2011). Similar performances were attained with didecyl dimethyl ammonium and trioctylmethylammonium chlorides (Yang et al., 2015), but none of them was tested on an ore.

#### Collector Mixtures

**Figure 9:**
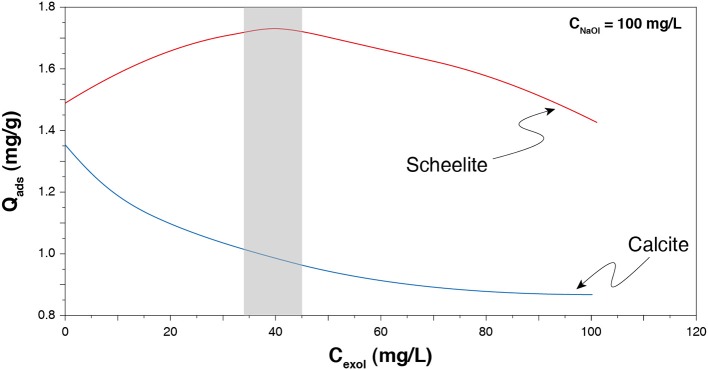
Effect of the quantity of an alcohol reagent (Exol-B) on the sodium oleate adsorption onto scheelite and calcite. Adapted from Filippov and Filippova (2006). The optimum zone is displayed in gray, which roughly corresponds to a 2.5:1 mass ratio.

To increase selectivity between scheelite and the other calcium minerals, one can use collector mixtures, including anionic/anionic, cationic/non-ionic, and anionic/non-ionic reagent mixtures. In particular, the use of a mixture of sodium oleate and fatty alcohols, i.e., non-ionic reagents, provided interesting performances for gypsum separation from a gangue comprising calcium minerals (Filippova et al., 2014). A similar mixture, involving sodium oleate and a fatty alcohol, conducted to an efficient flotation separation of scheelite from calcite (Filippov et al., 1993; Filippov and Filippova, 2006). It is assumed that mixing ionic and non-ionic reagents allows to reduce the electrostatic repulsions existing between the ionic groups of anionic collectors by introducing, in the adsorption layer, non-ionic compounds. These latter are supposed to adsorb on the cationic sites by means of electrostatic interactions and/or hydrogen bonding. In addition, while reducing the polar repulsions, they also maintain significant hydrophobic chain–chain interactions inside the adsorption layer (Filippov et al., 1993, 2012, 2019; Filippov and Filippova, 2006; Filippova et al., 2014) since they are composed of aliphatic chains of 10 to 16 carbon atoms. They induce higher quantities of collector adsorbed onto a mineral from another (Figure 9), due to a better organization of the adsorption layer. All the above mentioned studies clearly noticed significant variations in flotation selectivity and/or recoveries of various minerals. Similar results were observed using a combination of sodium oleate and polyoxyethyiene reagents (Chen C. et al., 2017; Chen et al., 2018).

**Figure 10:**
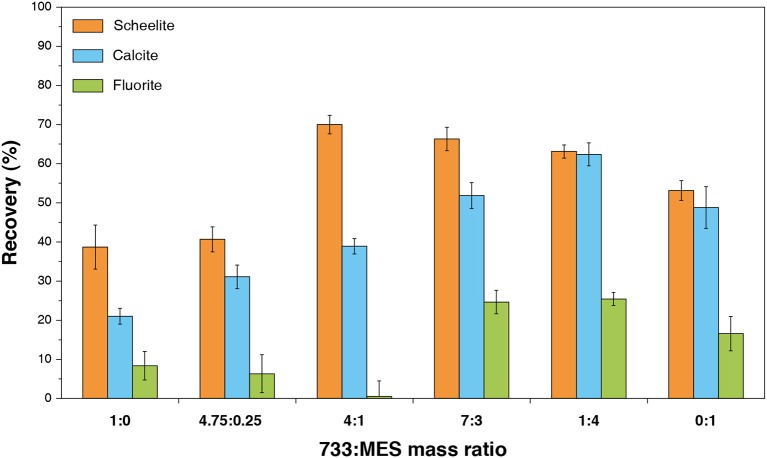
Effect of mass ratio of a fatty acid collector combined with methyl ester sulfonate on flotation behavior of scheelite, fluorite, and calcite. Adapted from Gao et al. (2015a) with permission of Elsevier.

Besides, a similar methodology was applied for anionic/anionic reagent mixtures by Yin and Wang (2014), who blended BHA and sodium oleate and observed an increase in the scheelite recovery, without yet performing any test on an ore (Yin and Wang, 2014). Another mixture of two different anionic collectors was studied recently: Gao et al. (2015a) blended a sulfonate with a fatty acid-based mixture with interesting results in terms of scheelite/fluorite selectivity (Figure 10). They obtained a concentrate assaying 65.8% WO_3_ with 66.0% WO_3_ recovery. This mixture not only improved the selectivity compared to sole sodium soap but also reduced the quantity of sodium soap and water glass required, with, finally, a high tolerance to water hardness (Gao et al., 2015a). Furthermore, recently, some authors demonstrated that mixing two molecules with the same polar group but different aliphatic chains can modulate the selectivity in flotation, particularly between scheelite and fluorite (Filippov et al., 2018). This achievement was attained by introducing saturated fatty acids (palmitate) in the unsaturated fatty acid-based formulations, which allowed increasing significantly the WO_3_ grade in the flotation concentrates. It demonstrates the importance of the chain geometry in the adsorption selectivity since this latter can be led by the auto-organization of the adsorption layer.

### Depressants for Common Gangue Minerals

According to the previous part, the most commonly used collectors for scheelite flotation are anionic reagents, mainly the carboxylates. Owing to their anionic groups, they all suffer from a significant lack of selectivity between the calcium salts, namely, scheelite, fluorite, apatite, and calcite. Therefore, the use of depressants is necessary to attain an acceptable selectivity in favor of scheelite. In particular, most collectors can afford a good selectivity between scheelite and a given mineral, while tungsten skarns generally comprise a dozen of minerals. The combination of a collector with a depressant is, therefore, mandatory to reach an acceptable separation between scheelite and several gangue minerals.

#### Sodium Silicate

**Figure 11:**
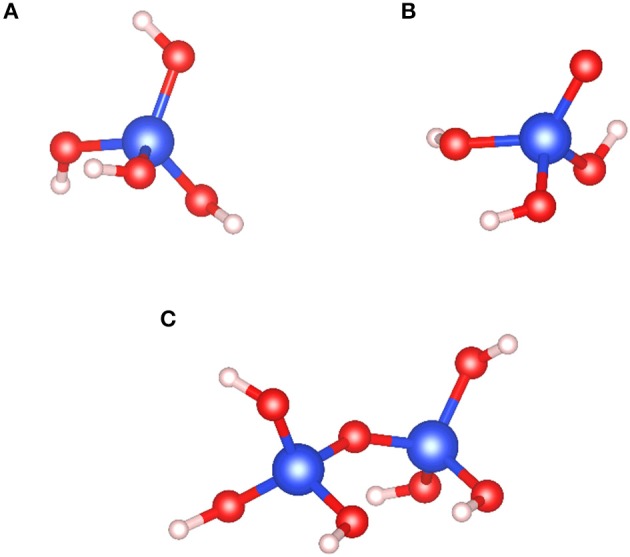
Most dominant forms of Na_2_SiO_3_ in aqueous solutions for realistic froth flotation conditions: **(A)** acidic monomer, Si(OH)_4_, **(B)** basic monomer, ${\text{SiO}(\text{OH})}_{3}^{-}$, and **(C)** acidic dimer, Si_2_O(OH)_6_. The blue, red, and white balls represent the silicon, oxygen, and hydrogen atoms, respectively.

Sodium silicate (Na_2_SiO_3_) is one of the most common reagents used in the froth flotation process (Leja, 1981). This environment-friendly reagent is a very efficient dispersant since it decreases the particle–particle interactions. Moreover, it is known to adsorb with a high affinity on the gangue mineral surfaces, mostly silicates, and therefore, it constitutes a strong depressant in the froth flotation process. Over the past decades, Na_2_SiO_3_ has been successfully used in the flotation separation of rare earth minerals (Filippov et al., 2016), zinc minerals (Ejtemaei et al., 2012), iron minerals (Rao et al., 2011), scheelite (CaWO_4_) (Kupka and Rudolph, 2018a; Yang, 2018; Foucaud et al., 2019c), fluorite (Song et al., 2006; Zhou et al., 2013), apatite (Qi et al., 1993; Sis and Chander, 2003), and many other minerals. The behavior of Na_2_SiO_3_ in aqueous solutions have been intensively investigated: depending on the pH of the solution and the total silica concentration, the silica tetrahedral (SiO_4_) can be under ${\text{SiO}}_{4}^{4-}$, SiO_3_(OH)^3−^, SiO_2_${\left(\text{OH}\right)}_{2}^{2-}$, ${\text{SiO}(\text{OH})}_{3}^{-}$, and Si(OH)_4_ forms (Engelhardt et al., 1975; Marinakis, 1980; Marinakis and Shergold, 1985; Bass and Turner, 1997; Halasz et al., 2007; Jansson et al., 2015). Furthermore, the pH, total silica concentration, and SiO_2_:Na_2_O ratio affect the polymerization degree of silica tetrahedra, a high ratio inducing a high polymerization degree (Lentz, 1964; Marinakis, 1980; Bass and Turner, 1997; Dimas et al., 2009; Nordström et al., 2011; Jansson et al., 2015). In the froth flotation process, according to the SiO_2_:Na_2_O ratio and the total silica concentrations commonly used, the main species of Na_2_SiO_3_ are Si(OH)_4_ for pH < 9.4 and ${\text{SiO}(\text{OH})}_{3}^{-}$ for 9.4 < pH < 12.6 (Marinakis, 1980; Han et al., 2016) (Figure 11). Also, the polymerization degree depends strongly on the ${\text{SiO}(\text{OH})}_{3}^{-}$ and SiO_2_(OH)${\left(\text{OH}\right)}_{2}^{2-}$ concentrations in solution (Roller and Ervin, 1940; Marinakis, 1980). Most of the flotation processes worldwide are performed at pH 7–11, at which the silica monomers are dominant, under both Si(OH)_4_ and ${\text{SiO}(\text{OH})}_{3}^{-}$ forms.

The above-mentioned silica species in aqueous solution are assumed to interact strongly with the surface cations, i.e., Ca^2+^ in the case of calcium salts. Depending on the surface calcium speciation (Ca or Ca–OH) and the most dominant silica species in solution, silica adsorption can result in the formation of Ca–Si(OH)_4_ or Ca–O–Si(OH)_3_ on the surface (Marinakis, 1980; Marinakis and Shergold, 1985). However, various adsorption mechanisms have been suggested, including the physical adsorption of silica gel and water glass (Cheng et al., 1963), silicate ions (Glembotskii and Uvarov, 1964; Fuerstenau et al., 1972), colloidal silica, and polymeric silicic acid (Nikiforov and Skobeev, 1968) apart from chemisorptions (Fuerstenau et al., 1972; Marinakis, 1980; Marinakis and Shergold, 1985). Besides, the soda amount as well as the age of the Na_2_SiO_3_ solution is likely to play a role in the depressing effect of Na_2_SiO_3_ (Berlinskii, 1962; Marinakis, 1980; Marinakis and Shergold, 1985). Recently, the adsorption of silica species onto fluorite, an archetypal calcium salt, has been investigated by DFT calculations (Foucaud et al., 2019d) the acidic form of the monomers and the dimers, Si(OH)_4_ and Si_2_O(OH)_6_, respectively, physisorbs onto surface calcium atoms, while the anionic form, namely, ${\text{SiO}(\text{OH})}_{3}^{-}$, chemisorbs onto surface calcium atoms, eventually substituting a fluorine surface atom. Also, the monomers adsorb with a considerable higher adsorption energy compared to the dimers (Foucaud et al., 2019d), which could explain the high depressing effect of acidified water glass (AWG). This reagent corresponds to sodium silicate treated with acid (sulfuric, hydrochloric, etc.) prior to its use in flotation. Over the past years, some authors demonstrated a high efficiency of AWG for depressing gangue minerals, in particular, calcite, significantly better than classical sodium silicate (Zhou et al., 2013; Bo et al., 2015; Dong et al., 2018; Kupka et al., 2019). According to these authors, this higher efficiency could be linked to higher amounts of depolymerized silica species (i.e., monomers) when sodium silicate is acidified (Zhou et al., 2013; Bo et al., 2015; Dong et al., 2018; Kupka et al., 2019).

#### Sodium Carbonate

Sodium carbonate (Na_2_CO_3_) is mostly described as a buffering pH modifier (Bulatovic, 2010) as well as a pulp dispersant (Kupka and Rudolph, 2018b), and eventually as a depressant (Zheng and Smith, 1997). Some authors highlighted positive synergistic effects when Na_2_SiO_3_ is used in flotation systems where the pH is controlled beforehand by Na_2_CO_3_ (Agar, 1984; Kupka and Rudolph, 2018a; Foucaud et al., 2019c). In other terms, the prior treatment of the minerals by Na_2_CO_3_ results in a higher efficiency of Na_2_SiO_3_ in terms of gangue mineral depression, which provides a better flotation selectivity and, therefore, considerably higher metal grades in the flotation concentrates (Martins and Amarante, 2013; Kupka and Rudolph, 2018b). Recently, Kupka and Rudolph (2018b) suggested that fluorite would be first depressed followed by calcite, silicates, and scheelite when Na_2_CO_3_ dosage is increased, with negligible dependence on the pH and the other pH modifiers used (Kupka and Rudolph, 2018b). The effect of Na_2_CO_3_ can be related to the precipitation of calcium ions in suspension (Bahr et al., 1968; Kupka and Rudolph, 2018b) as well as a surface carbonation of the calcium minerals (Bahr et al., 1968; Miller and Hiskey, 1972; Rahimi et al., 2017; Foucaud et al., 2019d). Indeed, the precipitation of calcium ions probably decreases the gangue mineral activation induced by their adsorption (Kupka and Rudolph, 2018b). Besides, the formation of calcium carbonate onto gangue mineral surfaces (silicates, apatite, fluorite, etc.), demonstrated by spectroscopic studies on fluorite (Foucaud et al., 2019d) results in a better depression of these minerals by Na_2_SiO_3_, which is known to strongly depress calcite (Filippov et al., 2018; Kupka and Rudolph, 2018b).

The addition of 1,150 g/t of sodium carbonate prior to the addition of sodium silicate (1,220 g/t) allowed to reach 11.2% WO_3_ with 95% recovery from a flotation feed containing 1.09% WO_3_ (Filippov et al., 2018). The synergistic effect highlighted is linked to the carbonation of the fluorite surfaces, calcite being well-depressed by sodium silicate. The combination of depressants was optimized to create the best conditions to estimate as well as possible the influence of the collector on the selectivity of flotation. Notably, *ab initio* calculations substantiated recent experimental investigations, which demonstrated that those synergistic effects are related to an acid–base reaction on the surface between the pre-adsorbed sodium carbonate layer and the silica species, added after (Foucaud et al., 2019d).

#### Metallic Salts

**Figure 12:**
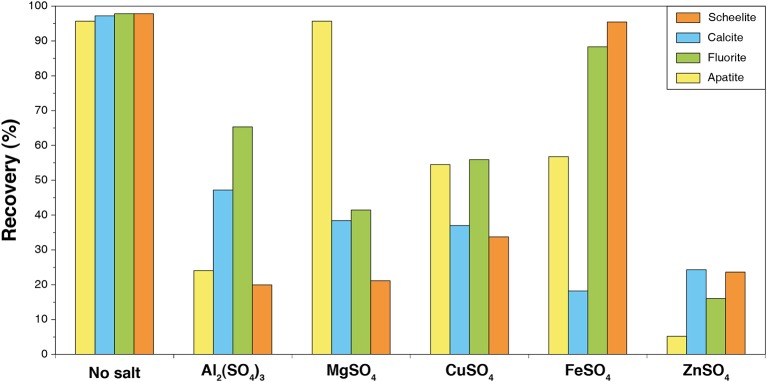
Combined effect of polyvalent metallic cations (300 g/t) and Na_2_SiO_3_ (1.5 kg/t) on the flotation recovery of calcium salts. Reproduced from Hanna and Somasundaran (1976) with permission of SME. Tests were performed with 2 kg/t Na_2_CO_3_ and 100 g/t oleic acid at pH 9.1–9.5.

Studies have demonstrated that the addition of metallic cations, such as Fe^3+^, Al^3+^, Pb^2+^, Zn^2+^, etc. in the flotation process allows to modulate the selectivity of the separation (Abeidu, 1973; Mercade, 1975; Hanna and Somasundaran, 1976; Detienne, 1978; Patil and Nayak, 1985; Oliveira and Sampaio, 1988; Schubert et al., 1990; Raatz, 1992; Feng et al., 2017) (see Figure 12). However, using sole metallic salts results in a limited improvement of the flotation performances (Hiçyìlmaz et al., 1993). Combined with Na_2_SiO_3_, these cations are assumed to form strongly hydrophilic hydrosols, which depress gangue minerals efficiently (Changgen and Yongxin, 1983). In particular, Patil and Nayak (1985) efficiently depressed calcite using a hydrosol composed of Na_2_SiO_3_ and ferrous sulfate with no negative impact on scheelite (Patil and Nayak, 1985). Interestingly, they noted an increase in the flotation recovery when the two reagents were added simultaneously. The use of iron salts combined with Na_2_SiO_3_ systematically conducted to an increase in the flotation selectivity in favor of scheelite (Dean and Schack, 1964; Foucaud et al., 2019c), which could be attributed to the specific adsorption of iron compounds (mostly hydroxides) on scheelite along with a strong depression of calcite by Na_2_SiO_3_. Nevertheless, the mechanisms involved in the depression/activation phenomena observed when Na_2_SiO_3_ is combined with metallic cations is still poorly understood.

#### Organic Molecules

Moreover, organic polymers such as tannins, carboxymethyl cellulose, and starch (Ozcan and Bulutcu, 1993; Rutledge and Anderson, 2015; Liu et al., 2016; Wang et al., 2018a) as well as chelating agents, e.g., citric acid (Bulatovic, 2015; Gao et al., 2016a), are known to depress calcium-bearing minerals in some cases. In particular, starch is widely known to strongly depress silicate minerals and calcite (Bulatovic, 2010), as well as quebracho and carboxymethyl cellulose (Wang et al., 2018a). Classical organic molecules were tested on a typical tungsten skarn without affording an acceptable selectivity between scheelite and the gangue minerals, mainly the calcium salts (Foucaud et al., 2019c). Nonetheless, many studies have been recently conducted to investigate the use of new organic depressants for the selective flotation of scheelite. Interestingly, Chen et al. (2017a) attained a satisfactory selectivity between scheelite and fluorite/calcite using sodium alginate as depressant and fatty acids as collector. They demonstrated that sodium alginate chemisorbs selectively onto fluorite and calcite, therefore preventing the adsorption of sodium oleate onto those minerals without affecting scheelite (Chen et al., 2017a). Similar performances were obtained using dextran sulfate sodium, which adsorbs selectively onto calcite and fluorite surfaces and, hence, increases significantly the flotation selectivity (Chen et al., 2017b). Some depressants were successfully used to depress specifically a given gangue mineral such as calcium lignosulfonate for calcite (Feng et al., 2018) or sodium polyacrylate for fluorite (Zhang et al., 2017). Overall, it has been demonstrated that all these abovementioned organic molecules adsorb by interactions between their polar groups (–OH or –COOH) and the surface cations, mainly Ca^2+^ (Somasundaran, 1969; Filho et al., 2000; Wang and Somasundaran, 2005; Filippov et al., 2013). These interactions can be of various types, including chemisorption of the –COO^−^ group onto calcium atoms, physisorption of the –OH or –COOH groups onto the same cations, or hydrogen bonding between the –OH or –COOH groups and hydroxylated surface cations [Ca(OH)^+^]. The selectivity obtained in the depression is most probably related to the steric hindrance of those organic molecules and the correlations between their functional groups (–OH or –COOH) and the surface cationic sites.

#### Phosphate Derivatives

Phosphate derivatives such as sodium hexametaphosphate [(NaPO_3_)_6_], sodium phosphate (Na_3_PO_4_), sodium pyrophosphate (Na_4_P_2_O_7_), and 1-hydroxyethylidene-1,1-diphosphonic acid (HEDP) (Wang et al., 2018b) are also widely used inorganic depressants for scheelite flotation from calcite and fluorite (Yongxin and Changgen, 1983; Bel'kova et al., 1993; Bulatovic, 2015; Liu et al., 2016). The depressing effect of phosphate derivatives has been extensively discussed, with, however, contradictory results (Kupka and Rudolph, 2018a). Indeed, some authors successfully used phosphate derivatives to depress calcite and fluorite in scheelite flotation (Changgen and Yongxin, 1983; Yongxin and Changgen, 1983), while other researchers employed them to depress apatite in fluorite flotation (Bulatovic, 2015) or in scheelite flotation (Bel'kova et al., 1993). Phosphate derivatives are known to chelate Ca^2+^ ions in solution leading to a surface depletion in terms of calcium ions faster for fluorite and calcite than for scheelite (Changgen and Yongxin, 1983). However, Gao et al. (2018) rather suggested a chemisorption of phosphate derivatives onto surface calcium atoms (Gao et al., 2018), which exhibit a higher density and activity for calcite than for scheelite.

## The Selective Separation of Scheelite: A Utopia?

### A Low Separation Contrast in Flotation

Nowadays, a few routes exist for efficient industrial-scale scheelite flotation in the presence of gangue calcium salts and silicates. Though amine collectors demonstrated acceptable performances, they are known to float silicates due to their physisorption on negatively charged surfaces. Since silicates represent most of the gangue minerals in tungsten skarns, the use of amines is unsuited to attain acceptable WO_3_ grades. Another method consists of the utilization of hydroxamic acids, mainly benzohydroxamic acid, in combination with lead cations. Nevertheless, in the EU, benzene-based compounds are forbidden, and the industrial use of lead is strictly restricted, making this method impossible to apply. The last proofed and widely used route is the flotation with fatty acids as collectors, especially sodium oleate, which are very efficient, cheap, and environment friendly. Their high efficiency is attributed to the chemisorption of the carboxylate group onto surface calcium atoms (Hanumantha Rao and Forssberg, 1991; Foucaud et al., 2018b). However, tungsten skarns commonly display high amounts of calcium-bearing minerals, either calcium silicates or calcium semi-soluble salts (fluorite, apatite, calcite, and scheelite), these latter exhibiting similar surface properties (Table 2). The flotation separation of scheelite from fluorite, apatite, and calcite has been intensively investigated over the past decades and remains, in the twenty first century, one of the most challenging problems in the froth flotation field.

**Table 2:** Calculated calcic site densities and surface Ca–Ca distance on the main cleavage planes for each studied mineral.

**Mineral**	**Cleavage planes (according to literature)**	**Ca–Ca distance (Å)**	**d(Ca^**2+**^) (Ca/Å^2^)**
Scheelite	(112) (Hu et al., 2012; Gao et al., 2013) (001) (Hu et al., 2012; Gao et al., 2013)	3.87 5.22	4.01 × 10^−2^ 3.67 × 10^−2^
Fluorite	(111) (Parks and Barker, 1977)	3.86	7.75 × 10^−2^
Apatite	(001) (Chang et al., 1996)	3.96	4.58 × 10^−2^
Calcite	(101) (Chang et al., 1996) (104) (Chang et al., 1996)	4.05 4.05	3.38 × 10^−2^ 4.58 × 10^−2^

### The Petrov Process

In the early 1940s, the tungsten demand was considerable for its wide range of military applications. Hence, the exploitation of tungsten skarns appeared crucial for the economic independence of the largest powers, including the USSR. Even then, the processing of tungsten skarns was difficult due to complex gangues comprising minerals with similar surface properties to those of scheelite. To face this issue, a method, developed by Petrov, allowed to increase significantly the selectivity (Petrov, 1940) and, therefore, the WO_3_ grade of the concentrates. It is based on the heating of the pulp to 80–100°C in the presence of considerable concentrations of Na_2_SiO_3_ (2–4%) after a roughing stage conducted with a classical process, i.e., sodium carbonate to control the pH, sodium silicate to depress the gangue minerals, and sodium oleate to collect scheelite. In this scheme, the rougher flotation concentrate comprises significant amounts of scheelite along with calcite, fluorite, and apatite. After heating, a cleaning stage is performed and usually allows increasing significantly the WO_3_ grade of the flotation concentrate, to attain WO_3_ grades that can be processed by hydrometallurgy. The surface physico-chemical phenomena at play during the Petrov process have never been understood and are still poorly known. Nonetheless, this process remains one of the most used processes for scheelite processing worldwide since it provides satisfactory selectivity after the roughing stage. The rejection of calcite, fluorite, and apatite from the flotation cleaning concentrates can be very high, affording acceptable WO_3_ grades for hydrometallurgy. Recently, some authors tried to substitute this process, which can be described as energy consuming considering the high temperatures that are required, by “greener” methods. This substitution is based on two main approaches: the development of specific depressants for gangue minerals, mostly the calcium salts, and the development of more specific collectors for scheelite. However, this substitution is very difficult since very few reagent formulations (depressants/collectors) exhibit satisfactory performances for all the gangue minerals, including the calcium salts (calcite, fluorite, apatite). Hence, avoiding the Petrov process depends on the product requirements existing on the scheelite concentrates for the hydrometallurgy subsequent stage and also on the contained gangue minerals. Nowadays, most Chinese and Russian mines still use the Petrov process for the industrial beneficiation of scheelite (Bernhart, 2015; Han et al., 2017) since it represents one of the only viable ways to attain the 60–65% WO_3_ required for the scheelite concentrates. Without this process, the common WO_3_ grades displayed, even after several cleaning stages, are as low as 30% WO_3_ with, moreover, unacceptable recoveries.

### Green Methods at Development

Flotation separation of calcium minerals with fatty acids is difficult: their chemisorption onto the surface calcium atoms along with the similar surface properties between the minerals generally (Table 2) induce a non-selective flotation. Nonetheless, different solutions can be undertaken to improve the elimination of calcium-bearing minerals during the mineral processing stage while maintaining a high level of environment friendliness:

(1) The minerals commonly contained in tungsten skarns exhibit a large range of specific gravities: 2.7 for calcite, 3.2 for fluorite and apatite, and 6.1 for scheelite, while silicates have specific gravities between 2.6 and 3.7, depending on the geological context. Hence, it allows one to perform a gravity separation to eliminate the minerals that are problematic in flotation with fatty acids, mostly the calcium-bearing semi-soluble salts (calcite, apatite, and fluorite). Nevertheless, the fine liberation size makes it difficult to process these ores by classical gravity separation methods, e.g., shaking tables, jigs, or spirals (Wells, 1991; Blazy and Joussemet, 2011; Das and Sarkar, 2018). Recently, Falcon concentrators, specifically designed for fine particle gravity processing, have been used successfully to achieve an efficient gravity separation on skarn ores (Foucaud et al., 2019b,e). Consistently, they afford a good elimination of the calcium salts that are problematic in flotation as well as fine particles (Dehaine et al., 2019), improving significantly the subsequent flotation stage.
(2) Additionally, skarns commonly contain iron, which is found within the ferromagnesian silicates of the protolith. After the thermometamorphism stage, iron is often included in the newly formed dense silicates such as vesuvianite, epidote, and garnets. Owing to the iron they contain, these silicates, which are denser compared to the non-ferromagnesian silicates, exhibit a significant magnetic susceptibility, ranking them in the paramagnetic mineral class. Hence, a high intensity magnetic separation is possible to eliminate these minerals that can represent high amounts in the tungsten skarns. It can allow reducing the amount of dense minerals going to the flotation stage after the Falcon gravity separation.
(3) In flotation, depressants can be added to enhance the separation differential between scheelite and the troublemaking minerals. Nevertheless, although silicates are quite easily depressed, very few depressants afford a satisfactory selectivity between scheelite and fluorite, apatite, and calcite. If some of them depress efficiently a specific gangue mineral, none can depress most gangue minerals without impacting significantly scheelite. In particular, the flotation separation between scheelite and fluorite has remained an industrial and a scientific challenge that has not been yet issued by the use of specific depressants. Hence, intensive developments should be done to find efficient and environmental friendly depressing conditions as well as to gain understanding in the adsorption mechanisms of the selected depressants onto calcium minerals. In most studies, the best depressing conditions are afforded by the use of the combination between sodium carbonate and sodium silicate.
(4) Considering the similar surface properties between scheelite and the other calcium minerals, the modulation of flotation collectors appears very difficult to separate scheelite from the other calcium minerals. According to recent developments, the flotation selectivity regarding the above-mentioned gangue minerals can be enhanced using collector mixtures (anionic/anionic or anionic/non-ionic), which exhibited satisfactory results in terms of flotation selectivity. Therefore, these collector mixtures should be intensively investigated to modulate the flotation selectivity in favor of scheelite and to gain understanding in the synergistic effects that they can exhibit.

The four above mentioned points should be thoroughly investigated to develop an efficient, adaptable, integrated, and environmental friendly process for a tungsten skarn. Besides, the froth flotation process is led by the adsorption of various reagents, organic or mineral, at the liquid/solid interface, with the objective of selectively rendering the target mineral(s) hydrophobic to recover it in the froth phase. Collectors, which contain a polar group and a non-polar aliphatic chain, can be adapted in terms of chain length, unsaturation, and ramification, as well as functionalized polar group. Moreover, all reagents can be combined to improve the flotation performances [selectivity or target mineral(s) recovery]. All these optimizations are difficult to investigate by experimental methods, and molecular modeling has become a powerful tool to gain understanding in the adsorption mechanisms of flotation reagents at the mineral surfaces (Foucaud et al., 2019a). In particular, *ab initio* atomistic simulations can be applied in the future to study the fundamentals of fatty acid adsorptions onto calcium minerals and to unravel the synergistic effects highlighted when different reagents are combined together (either depressants or collectors).

### With Helpful Perspectives Provided by Atomistic Simulations

Tungsten skarns are not the only ores displaying complex gangues. Indeed, over the past decades, the gangue complexness has been constantly increasing, which means that the gangue minerals are being more and more difficult to separate from the target mineral(s). This decrease in the separation contrast in flotation for most ores, including tungsten skarns, can, however, be overcome by increasing the flotation performances, i.e., the flotation selectivity and recovery. Those two key indexes mostly depend on the adsorption of flotation reagents (depressants and collectors) onto target(s) and gangue minerals. Therefore, it is of paramount interest to investigate thoroughly the molecular mechanisms involved in the adsorption of flotation reagents since they are still poorly described. Atomistic simulations such as quantum mechanic simulations or classical molecular dynamic simulations have been playing this role for several years (Badawi et al., 2011; Souvi et al., 2017; Hessou et al., 2018; Berro et al., 2019). The different molecular simulation methods existing nowadays in theoretical physics and chemistry, which can be applied to froth flotation, have been critically reviewed recently (Foucaud et al., 2019a). In particular, these methods contribute to improve the comprehension of the synergistic effects that can be significantly displayed when flotation reagents are used in combination (depressants, collectors, or both). Furthermore, they are involved in the development of novel reagent formulations and/or combinations based on the computed adsorption energies (Chehaibou et al., 2019; Rocca et al., 2019).

When applied to tungsten skarn ores, atomistic simulations can allow to:

(1) Compare the reactivity of the surfaces of the different minerals (mostly scheelite and fluorite, the main troublemaking mineral) toward water molecules, since the froth flotation process is performed in water. In particular, the water molecules adsorb onto the surface (111) of fluorite with around −55 kJ.mol^−1^ (Foucaud et al., 2018a), which is low compared to the adsorption energies of water reported on the most exposed surfaces of scheelite (Cooper and de Leeuw, 2003; Gao et al., 2013).
(2) Investigate the adsorption mechanisms of flotation reagents onto oxide minerals. Anionic collectors are traditionally used for oxide mineral flotation and require first principle simulations such as DFT to describe accurately the chemisorption processes. Besides, these regents can also be considered under their protonated, i.e., neutral form, to avoid the problem of bond creation. In this case, classical molecular dynamic simulations can be conducted to investigate the molecular mechanisms involved in the adsorption of these reagents. For instance, the adsorption mechanisms of carboxylates onto scheelite (Cooper and de Leeuw, 2004; Zhao et al., 2013; Yin et al., 2015) and fluorite have been investigated albeit a lot still has to be done.
(3) Understand and optimize the synergistic effects between reagents of the same sort (collectors, depressants) as well as between collectors and reagents. Novel reagent formulations can be developed to increase either the flotation selectivity or the flotation recovery.

### The Processes Currently Used in the Industry

At the moment, a few mines in the world process scheelite-bearing ores. Owing to the abovementioned difficulties for scheelite beneficiation, several options are currently or have recently been used in the tungsten extraction industry. First, some mines, still in operation in 2020, prefer the use of gravity separation only when it is possible. It is the case for the Nui Phao Vietnamese mine (7% of the global W production) and for the Los Santos Spanish mine (1% of the global W production): they just perform talc and/or sulfide flotation stage before or after the use of classical gravity separations such as shaking tables and spirals (Ngoc, 2016). However, these latter are rarely possible considering the scheelite liberation size. Also, they generally lead to lower overall scheelite recoveries compared to flotation, and consequently, most scheelite mines prefer nowadays the use of classical flotation schemes. It is the case of the other scheelite mines worldwide, including the Vostok-2 Russian mine (3% of the global W production), the Mittersill Austrian mine (1–2% of the global W production), the Shizhuyuan Chinese mine (20% of the global W production), and most probably the other Chinese mines, although little information is available. Since 1940, Russian mines have systematically used the Petrov process for scheelite beneficiation, as is still the case in the Vostok-2 processing plant (Bernhart, 2015). First, after the sulfide flotation stage, a rougher scheelite flotation stage is performed using sodium carbonate to control the pH, sodium silicate to depress the gangue minerals, and oleic acid as the collector (Bernhart, 2015). The bulk concentrate, comprising scheelite along with calcite, fluorite, and apatite, undergoes the Petrov process to obtain, after two additional flotation stages, a scheelite concentrate assaying 53% WO_3_ with an overall WO_3_ recovery of 85% (Bernhart, 2015). In Shizhuyuan operations, the selectivity between scheelite and fluorite is increased by the use of a mixture of fatty acids and benzohydroxamic acid, both combined with lead nitrate (Han et al., 2017). Consistently, high amounts of sodium silicate are used to depress the gangue minerals, including fluorite and, unfortunately, scheelite. However, this development is not enough, and the flotation concentrate has to undergo the Petrov process to meet the product requirements in terms of WO_3_ grade (Han et al., 2017). Besides, in Mittersill, the use of the Petrov process is avoided since the subsequent metallurgical stage accepts lower WO_3_ grade in the scheelite concentrate. Hence, the Mittersill mine produces scheelite concentrate grade assaying only 32% WO_3_(Bernhart, 2015), owing to the dilution by calcite, apatite, fluorite, and other problematic calcium gangue minerals, which are still present in the concentrate. The same reagents (sodium silicate and fatty acids), widely used for scheelite flotation, are used in Mittersill operations and are responsible for the low selectivity between scheelite and calcium gangue minerals. The use of the Petrov process was also avoided in the Cantung Canadian formerly exploited scheelite mine, where gravity pre-concentration allowed rejecting the problematic gangue minerals prior to flotation (Delaney and Bakker, 2014) as recently been suggested for other European skarns (Foucaud et al., 2019b,e).

## Conclusion

The froth flotation of scheelite still remains, in the twenty first century, a strong scientific, industrial, and technical challenge. At the moment, very few options are suitable considering the complexness of the gangue and the fine liberation size of tungsten skarns as well as the environmental concerns. In this paper, the main collectors and depressants that can be used for scheelite flotation have been thoroughly reviewed with the global objective to attain acceptable selectivity and recovery. Although many studies exhibited interesting performance with various flotation reagents, the greenest, most efficient, and cheapest method for scheelite flotation is to use fatty acids as collectors with sodium silicate as depressant. However, this solution suffers from a crucial lack of selectivity regarding the calcium salts, namely, fluorite, calcite, and apatite, which generally represent significant amounts in tungsten skarns. Those minerals, mainly fluorite, are difficult to separate from scheelite and, therefore, dilute dramatically the scheelite concentrates. More selective reagents or fatty acid-based formulations, reviewed in the present work, can hence be used to separate fluorite from scheelite, and when this approach is limited, a combination with gravity separation can be necessary. Furthermore, the challenge of selectively floating scheelite can be issued by modulating the synergistic effects commonly observed when reagents are combined together (depressants/depressants, collectors/collectors, or depressants/collectors). Overall, the atomistic simulations can provide a significant help to gain understanding in the modulation of the flotation selectivity regarding scheelite. Their use in the flotation area is considerably increasing and will be, in the next years, a crucial tool to reach the flotation performances required for the processing of low-grade fine-grained complex ores. In particular, it can allow to unravel the synergistic effects observed empirically in flotation experiments and to gain understanding in the fundamentals of flotation reagent adsorption onto mineral surfaces.

## Author Contributions

YF, LF, IF, and MB designed the study and the content of this review. YF and MB drafted the paper. LV and IF supervised the work of YF. All authors contributed to manuscript corrections and approved the submitted version.

### Conflict of Interest

The authors declare that the research was conducted in the absence of any commercial or financial relationships that could be construed as a potential conflict of interest.
